# MicroRNA-181 as a prognostic biomarker for survival in acute myeloid leukemia: a meta-analysis

**DOI:** 10.18632/oncotarget.19195

**Published:** 2017-07-12

**Authors:** Qiang Guo, Junwen Luan, Ni Li, Zhen Zhang, Xiaoxiao Zhu, Lin Zhao, Ran Wei, Linlin Sun, Yin Shi, Xunqiang Yin, Na Ding, Guosheng Jiang, Xia Li

**Affiliations:** ^1^ Laboratory for TCM Immunology and Epigenetics, Institute of Basic Medicine, Shandong Academy of Medical Sciences, Jinan 250062, Shandong, China; ^2^ Muping Hospital of Traditional Chinese Medicine, Yantai 264100, Shandong, China; ^3^ School of Medicine and Life Sciences, University of Jinan-Shandong Academy of Medical Sciences, Jinan 250062, Shandong, China; ^4^ Shandong Institute of Scientific and Technical Information, Jinan 250101, Shandong, China

**Keywords:** acute myeloid leukemia, biomarker, microRNA-181, overall survival, prognosis

## Abstract

Accumulating evidence has indicated that microRNA-181 (miR-181) is dysregulated in hematological malignancies, and associates with the clinical outcomes. However, the association of miR-181 expression levels with acute myeloid leukemia (AML) remains inconclusive, as publications from different groups have reported contradictory results. In this manuscript, a meta-analysis was performed to assess the prognostic significance of miR-181 in AML patients. Eligible studies were retrieved from PubMed, Embase and Cochrane Library databases, and a total of 6 studies including 815 AML patients were included in the final analysis. Hazard ratios (HRs) and their corresponding 95% confidence intervals (CIs) were extracted and pooled to investigate the correlation between miR-181 and the survival of AML patients. Our results showed that elevated miR-181 expression was associated with increased survival in 395 American patients, and reduced survival in 325 Chinese patients. Both subgroup analyses and meta-regression indicated that the origin of AML patients contributed to the heterogeneity in the datasets evaluating the correlation between overall survival (OS) and miR-181. These results indicate that miR-181 can be used as a promising prognostic biomarker in AML patients, which may depend on the origin of patient population.

## INTRODUCTION

Acute myeloid leukemia (AML), one of the most common types of hematopoietic malignancy, is the leading cause of leukemia death in USA [1]. According to the statistics, the annual incidence rate of AML was 4.14 per 100,000 in USA (2009–2013) [1], and 4.39 per 100,000 in UK (2004–2013) [2]. There was no detailed prevalence of AML in China, but it was reported that the annual incidence rate of leukemia was 5.00 per 100,000 in China (2013) [3], and the annual incidence rates of AML was 1.35 per 100,000 in Nanjing (2003–2007) [4]. AML is more common in adults with the median age being 67 years old at diagnosis, and about 4% patients are children and adolescents [5]. With the development of chemotherapy and hematopoietic stem cell transplantation, the clinical outcomes of AML patients have been improved greatly in the past few decades [6, 7]. However, relapsed and refractory leukemia remain as the major causes of mortality in AML patients [8, 9]. Several criteria have been proposed to classify leukemia subtypes and predict prognosis, based on morphological features or different biomarkers identified via serology analysis, immunophenotyping or genetic profiling [10, 11]. These methods and criteria are important for the clinical diagnosis and treatment of leukemia [12]; however, one biomarker is not sufficient enough to distinguish leukemia subtypes for personalized therapeutic strategies. Therefore, it is of great importance to explore more reliable prognostic biomarkers and use them as combinations for accurate diagnosis and medical decision-making for AML patients.

MicroRNAs (miRNAs) are a class of highly conserved small noncoding RNAs (range from 18 to 24 nucleotides) in a variety of organisms, which regulate target gene expressions via degradation or inhibition of the target mRNAs translation upon binding to 3′-untranslated regions of target mRNAs [13–16]. Recently, accumulating investigations have identified the distinct expression patterns and biological functions of miRNAs [17–19], as well as their potential roles as diagnostic and prognostic biomarkers in leukemia [20, 21]. The microRNA-181 (miR-181) family includes 4 members (miR-181a, miR-181b, miR-181c and miR-181d), which are highly evolutionarily conserved across almost all vertebrates [22, 23]. Human miR-181a and miR-181b genes are located on chromosomes 1 (miR-181a1 and miR-181b1) and 9 (miR-181a2 and miR-181b2), whereas miR-181c and miR-181d genes are clustered closely on chromosome 19 [24]. A single nucleotide polymorphism (SNP), rs78086449, is existed in human pre-miRNA regions of miR-181b2 [25]. Recently, it has been demonstrated that the expression of miR-181 is related to the prognosis of AML patients [24, 26]. Li et al. showed that increased expression of miR-181a or miR-181b was significantly associated with longer overall survival (OS) in cytogenetically abnormal AML (CA-AML) patients [27], which was consistent with Schwind’s findings in cytogenetically normal AML (CN-AML) patients [28]. In contrast, Zhi et al. and Xiang et al. reported that high miR-181b expression was associated with poorer prognosis, as revealed by higher OS or reduced complete remission (CR), in AML patients [29, 30]. In addition, Butrym et al. also found that AML patients with lower expression of miR-181a showed longer survival rates [31]. The contradictory results among these studies made it difficult to assess the prognostic effect of miR-181 in AML patients.

To overcome the discrepancy and low reproducibility of individual studies evaluating prognostic value of miR-181 in AML patients, a meta-analysis was performed in the present study. Eligible studies on the roles of miR-181 in AML patients were identified and retrieved from databases of PubMed, Embase and Cochrane Library. Moreover, hazard ratios (HRs) and their corresponding 95% confidence intervals (CIs) were extracted from individual study and pooled together in overall meta-analysis to elucidate the prognostic value of miR-181 in AML patients. Our results indicate that miR-181 can be used as a prognostic biomarker in AML patients based on the origin of patient population.

## RESULTS

### Summary of analyzed studies

**Figure 1:**
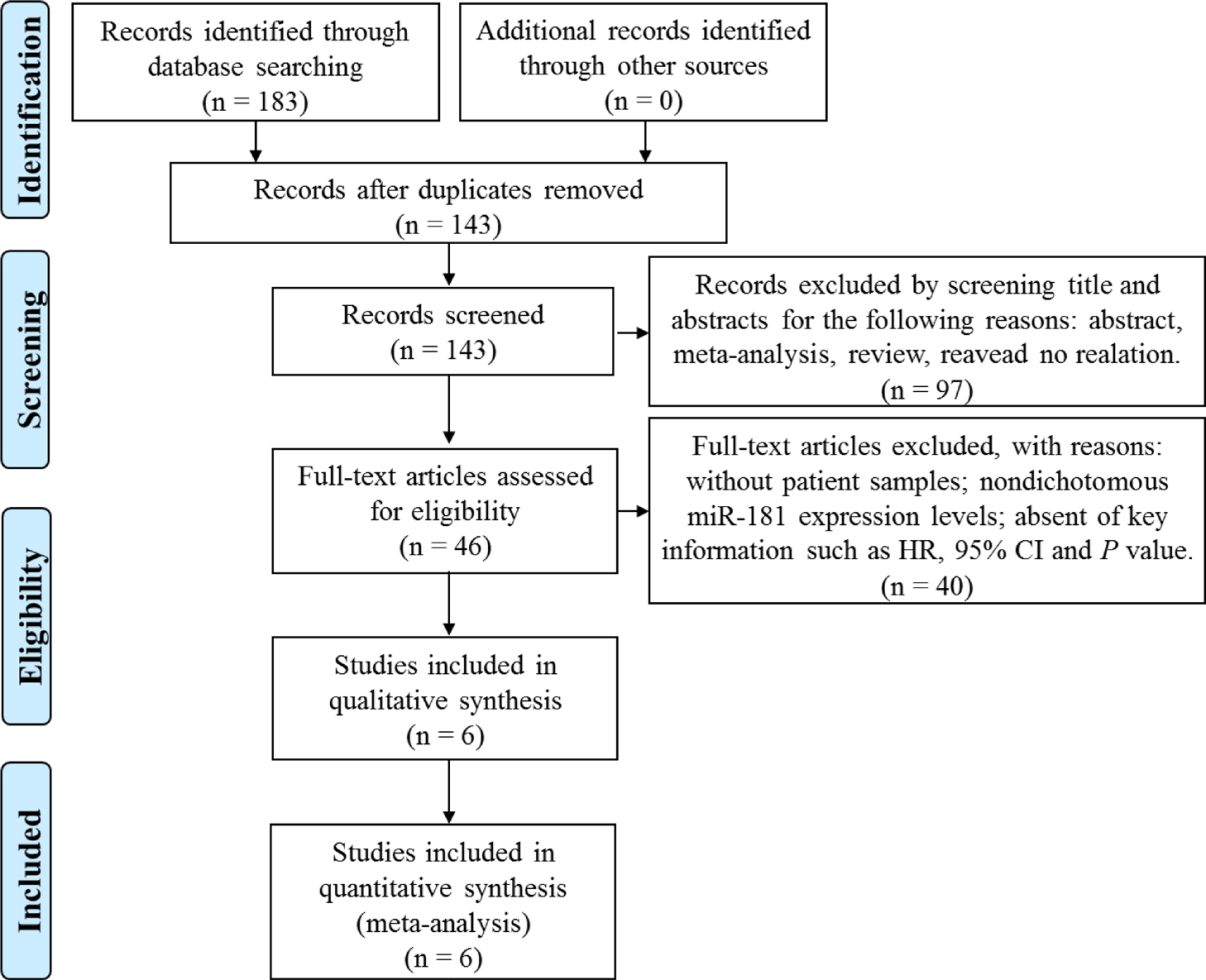
Flow diagram of the screening and selection process of studies

A flowchart of detailed searching process was illustrated in Figure 1. Using the three-step literature searching strategy, a total of 183 articles were initially identified from PubMed, Embase, and Cochrane Library databases. Following removal of 40 duplicate records, another 97 records that are conference abstracts, meta-analysis, reviews or non-relevant studies were excluded based on manual screening of the abstracts and titles. Full-text of the remaining 46 articles were further evaluated, and 6 articles were selected for the present meta-analysis based on our inclusion and exclusion criteria described in Methods. The public repositories including GEO, EBI ArrayExpress, and TCGA were also searched, but no eligible datasets were founded.

The main characteristics and basic information of eligible studies were listed in Table 1. Due to the inability to acquire information concerning the race and ethnicity of the patients, these studies were analyzed according to the country of the patients. A total of 815 patients diagnosed as AML, CA-AML, CN-AML in China [29, 30, 32], Poland [31] and USA [27, 28] were enrolled in these studies. The clinical data on these patients were shown in Supplementary Table 1. Four studies used quantitative PCR (qPCR) to measure the expression levels of miR-181 (TaqMan: 2 and SYBR Green PCR: 2), and another 2 studies employed the microarray method. Li et al. analyzed all members of miR-181 family, and investigated two independent populations as a training set and a validation set [27]. Schwind et al. examined miR-181a expression in 187 younger (< 60 years) adults with CN-AML and 122 CN-AML patients with FLT3-internal tandem duplication (FLT3-ITD) and/or NPM1wt haplotypes (≥ 60 years) [28]. Zhi et al. estimated the OS of AML patients based on the expression levels of miR-181b-5p (high versus low expression group) in a training set, a validation set, and the combined set [29], and data of the combined set were extracted for analysis herein. Among these eligible studies, the prognostic role of miR-181 was evaluated in 13 datasets for OS, 3 datasets for CR, 2 datasets for disease-free survival (DFS), and 2 datasets for relapse-free survival (RFS).

**Table 1:** Main characteristics of eligible studies

Author	Year	Origin of populations	Leukemia	Sample number	Specimen	miR-181	Detection method^a^	Cut-off value	Survival analysis	Outcome	Source of HR
Butrym et al.	2016	Poland	AML	95	Bone marrow	miR-181a	TaqMan	Mean	Multivariate analysis	OS	Kaplan-Meier curve
Li et al. (1)	2012	USA	CA-AML	33	Not reported	miR-181a, b, c, d	Microarray	Median	Multivariate analysis	OS	Reported
Li et al. (2)	2012	USA	CA-AML	53	Not reported	miR-181a, b, c, d	Microarray	Median	Multivariate analysis	OS	Reported
Liu et al.	2016	China	pediatric AML	27	Bone marrow	miR-181a	SYBR Green	Mean	Kaplan-Meier method	RFS	Kaplan-Meier curve
Schwind et al. (1)	2010	USA	CN-AML	187	Bone marrow	miR-181a	Microarray	Median	Multivariate analysis	OS, CR, DFS	Reported
Schwind et al. (2)	2010	USA	CN-AML with FLT3-ITD and/or NPM1wt	122	Bone marrow	miR-181a	Microarray	Median	Multivariate analysis	OS, CR, DFS	Reported
Xiang et al.	2013	China	AML	158	Bone marrow	miR-181b	SYBR Green	Median	Multivariate analysis	OS, CR, RFS	Reported
Zhi et al.	2013	China	AML	140	Serum	miR-181b	TaqMan	Median	Kaplan-Meier method	OS	Kaplan-Meier curve

AML: acute myeloid leukemia; CA-AML: cytogenetically abnormal acute myeloid leukemia; CN-AML: cytogenetically normal acute myeloid leukemia; HR, hazard ratio; OS: overall survival; CR: complete remission; DFS: disease-free survival; RFS: relapse-free survival. aDetection method: both TaqMan and SYBR Green are quantitative real-time PCR methods.

Study quality was assessed by the Newcastle-Ottawa Quality Assessment Scale (NOS), which comprises the following three parameters of quality: the selection, the comparability and the outcome [33]. All of the selected 6 studies scored 1 or higher on each of the 3 parameters, and total scores ranged from 6 to 8 (Table 2). The study quality is considered as high if the total score is greater than 5 [34]. Hence, these studies are of a relatively high quality and are included in the final analysis.

**Table 2:** Quality assessment based on the newcastle–ottawa quality assessment scale

Author	Year	Selection	Comparability	Outcome	Total score
Butrym et al.	2016	4	1	1	6
Li et al.	2012	4	2	1	7
Liu et al.	2016	4	1	1	6
Schwind et al.	2010	4	2	2	8
Xiang et al.	2013	4	1	1	6
Zhi et al.	2013	4	1	1	6

### Association of OS with miR-181 expression

**Figure 2:**
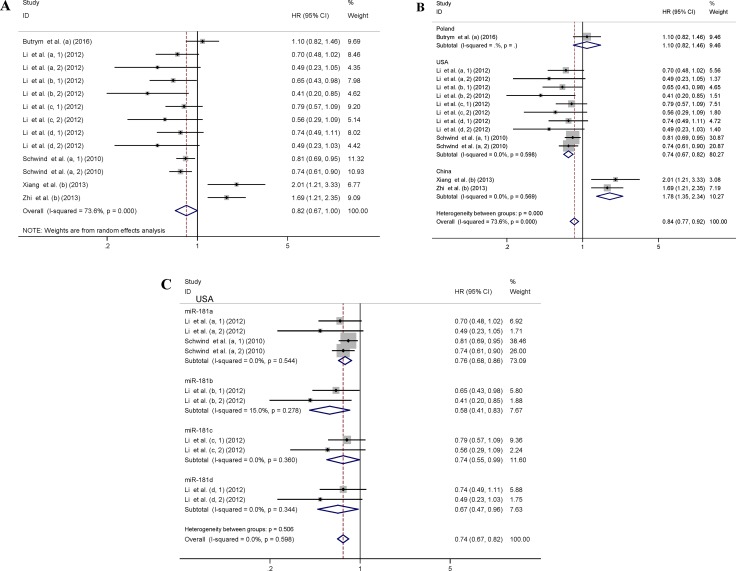
Forest plots of studies evaluating the pooled HR of elevated miR-181 levels for OS Forest plot of the relationship between miR-181 expression and OS using a random-effects model (**A**), by subgroup analysis based on origin of population using a fixed-effect model (**B**), by subgroup analysis based on members of miR-181 family in American patients using a fixed-effect model (**C**). The lower-case letters a, b, c, d represent that miR-181a, miR-181b, miR-181c and miR-181d were analyzed separately in the studies.

The main results of this meta-analysis were displayed in Figure 2. Thirteen datasets including 788 patients were investigated to understand the relationship between miR-181 expression and OS in AML patients. As some HRs in studies were not directly reported and could not be obtained from the authors, Kaplan-Meier curves were used to extract data [35, 36]. For these datasets, the pooled HR and its 95% CI were calculated by a random-effects model (Figure 2A). Furthermore, seven subgroup analyses of OS were performed through classifying patients based on origin of population, specimen, member of miR-181 family, detection method, cut-off value, survival analysis and source of HR (Table 3). Subgroup analysis by origin of population showed that high miR-181 levels were significantly associated with a favorable OS in American patients (pooled HR: 0.74, 95% CI: 0.67–0.82, *P* < 0.01), but suggested a worse OS in Chinese patients (pooled HR: 1.78, 95% CI: 1.35–2.34, *P* < 0.01) (Table 3 and Figure 2B). There was no significant difference in relationship between expression levels of individual miR-181 family member and OS in the subgroup analysis in American patients (Figure 2C). Since there were only miR-181a expression in Polish patients and only miR-181b expression in Chinese patients, the subgroup analysis based on members of miR-181 family was not performed in Polish and Chinese patients.

**Table 3:** Subgroup analyses of the relationship between miR-181 expression and OS

Subgroup	Number of datasets	Number of patients	Model^b^	Heterogeneity		Pooled HR (95% CI)	*P* value	Meta-regression^c^			
				*I*^2^ (%)	*P*-value			Tau^2^	Adj R^2^ (%)	*P* value	Adjusted *P* value
**All**	13	788	Random	73.6	< 0.001	0.82 (0.67, 1.00)					
**Origin of population**								0	100.00	0.000	0.002
Poland	1	95	Fixed			1.10 (0.82, 1.46)	0.53				
USA	10	395	Fixed	0.0	0.598	0.74 (0.67, 0.82)	< 0.01				
China	2	298	Fixed	0.0	0.569	1.78 (1.35, 2.34)	< 0.01				
**Specimen**								0.1523	-18.54	0.793	0.999
Bone marrow	4	562	Random	81.6	0.001	0.99 (0.74, 1.32)	0.94				
Not reported	8	86	Fixed	0.0	0.701	0.67 (0.56, 0.79)	< 0.01				
Serum	1	140	Fixed			1.69 (1.21, 2.35)	< 0.01				
**miR-181**								0.1436	-11.73	0.602	0.979
miR-181a	5	490	Fixed	45.0	0.122	0.80 (0.72, 0.90)	0.01				
miR-181b	4	384	Random	88.0	< 0.001	1.01 (0.52, 1.97)	0.98				
miR-181c	2	86	Fixed	0.0	0.360	0.74 (0.55, 0.99)	0.04				
miR-181d	2	86	Fixed	0.0	0.344	0.67 (0.47, 0.96)	0.03				
**Detection method**^a^								0.01704	86.74	0.002	0.016
TaqMan	2	235	Random	72.9	0.055	1.35 (0.88, 2.06)	0.17				
SYBR Green	1	158	Fixed			2.01 (1.21, 3.33)	< 0.01				
Microarray	10	395	Fixed	0.0	0.598	0.74 (0.67, 0.82)	0.01				
**Cut-off value**								0.1392	-8.29	0.457	Dropped^d^
Mean	1	95	Fixed			1.10 (0.82, 1.46)	0.53				
Median	12	693	Random	73.7	< 0.001	0.80 (0.64, 0.98)	0.03				
**Survival analysis**								0.06606	48.59	0.048	0.151
Multivariate analysis	12	648	Random	59.5	0.004	0.78 (0.65, 0.92)	< 0.01				
Kaplan-Meier method	1	140	Fixed			1.69 (1.21, 2.35)	< 0.01				
**Source of HR**								0.05805	54.83	0.035	0.081
Kaplan-Meier curve	2	235	Random	72.9	0.055	1.35 (0.88, 2.06)	0.17				
Reported	11	553	Random	54.2	0.016	0.74 (0.63, 0.88)	< 0.01				

HR, hazard ratio; CI, confidence interval. ^a^Detection method: both TaqMan and SYBR Green are quantitative real-time PCR methods. ^b^Model: Fixed, fixed-effect model; Random, random-effects model. ^c^Meta-regression was performed to explore possible explanations for heterogeneity with Monte Carlo permutation test (Permutations = 10,000). ^d^Source of HR dropped due to collinearity in meta-regression with Monte Carlo permutation test.

### Heterogeneity analysis

Obvious heterogeneity (*I*^2^ = 73.6%, *P* < 0.001) was discovered among the selected datasets evaluating the correlation between OS and miR-181 (Table 3 and Figure 2A). The higher significant heterogeneity was found in datasets with miR-181b (*I*^2^ = 88.0%, *P* < 0.001) and bone marrow specimen type (*I*^2^ = 81.6%, *P* = 0.001) (Table 3). However, the heterogeneity was significantly decreased in the subgroup analysis by origin of population. A meta-regression analysis was performed to investigate the sources of the heterogeneity in OS analysis group (Table 3), based on origin of population, specimen, member of miR-181 family, detection method, cut-off value, survival analysis and source of HR as covariates. All covariates were entered into the meta-regression model simultaneously, and the Monte Carlo permutation tests were performed for 10,000 times to acquire higher precision. We found that the origin of population (adjusted *P* = 0.002) and detection method (adjusted *P* = 0.016) together contributed to the heterogeneity to one degree or another (Table 3).

### Association of CR, DFS, and RFS with miR-181 expression

**Figure 3:**
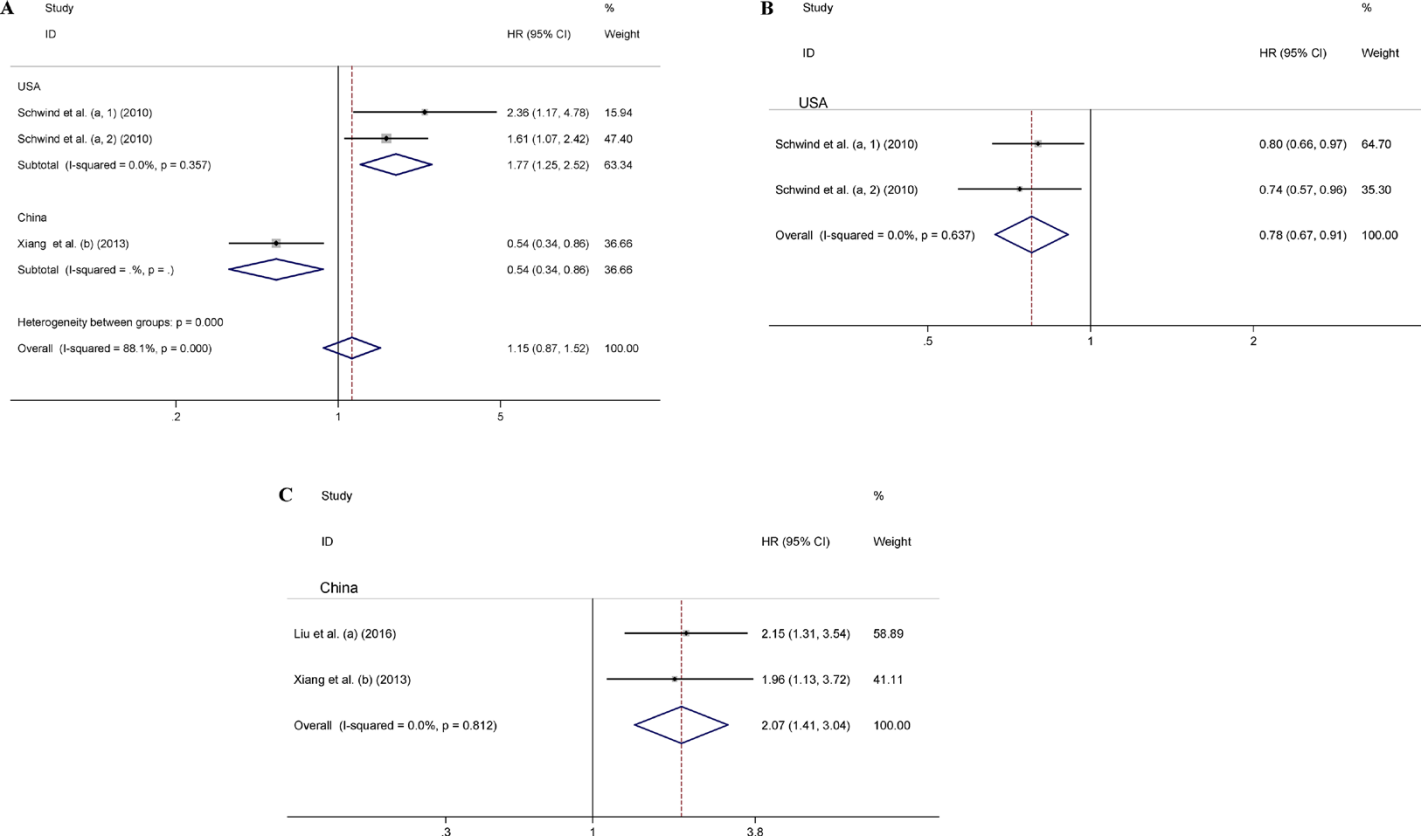
Forest plots of the relationship between miR-181 expression and CR (**A**), DFS (**B**), and RFS (**C**). The lower-case letters a, b represent that miR-181a and miR-181b were analyzed separately in the studies.

Three datasets including 467 patients investigated the prognostic role of miR-181 for CR (Figure 3A). A random-effects model was utilized to calculate the pooled HR and its 95% CI, due to the high heterogeneity among these datasets (*I*^2^ = 88.1%, *P* < 0.001). The meta-regression was not used for heterogeneity analysis of the CR, due to smaller size of the qualified datasets (< 10). Two datasets including 250 American patients evaluated the relationship between miR-181 and DFS (Figure 3B), and two datasets including 185 Chinese patients studied the relationship between miR-181 and RFS (Figure 3C). A fixed-effect model was applied to assess the association between miR-181 and DFS/RFS, respectively, since no heterogeneity was found among the datasets of each group. As origin of population in AML patients contributed to the heterogeneity in datasets evaluating the correlation between OS and miR-181, the datasets evaluating the correlation between CR, DFS, RFS and miR-181 were also investigated by subgroup analysis based on origin of population. Altogether, our results showed that high miR-181 level predicted favorable responses in terms of CR (pooled HR: 1.77, 95% CI: 1.25–2.52), DFS (pooled HR: 0.78, 95% CI: 0.67–0.91) in American AML patients (*P* < 0.01), and inferior outcomes with CR (HR: 0.54, 95% CI: 0.34–0.86), RFS (pooled HR: 2.07, 95% CI: 1.41–3.04) in Chinese AML patients (*P* < 0.01) (Figure 3). Collectively, our results support the prognostic prediction of miR-181 in AML patients based on the origin of population.

### Sensitivity analysis

**Figure 4:**
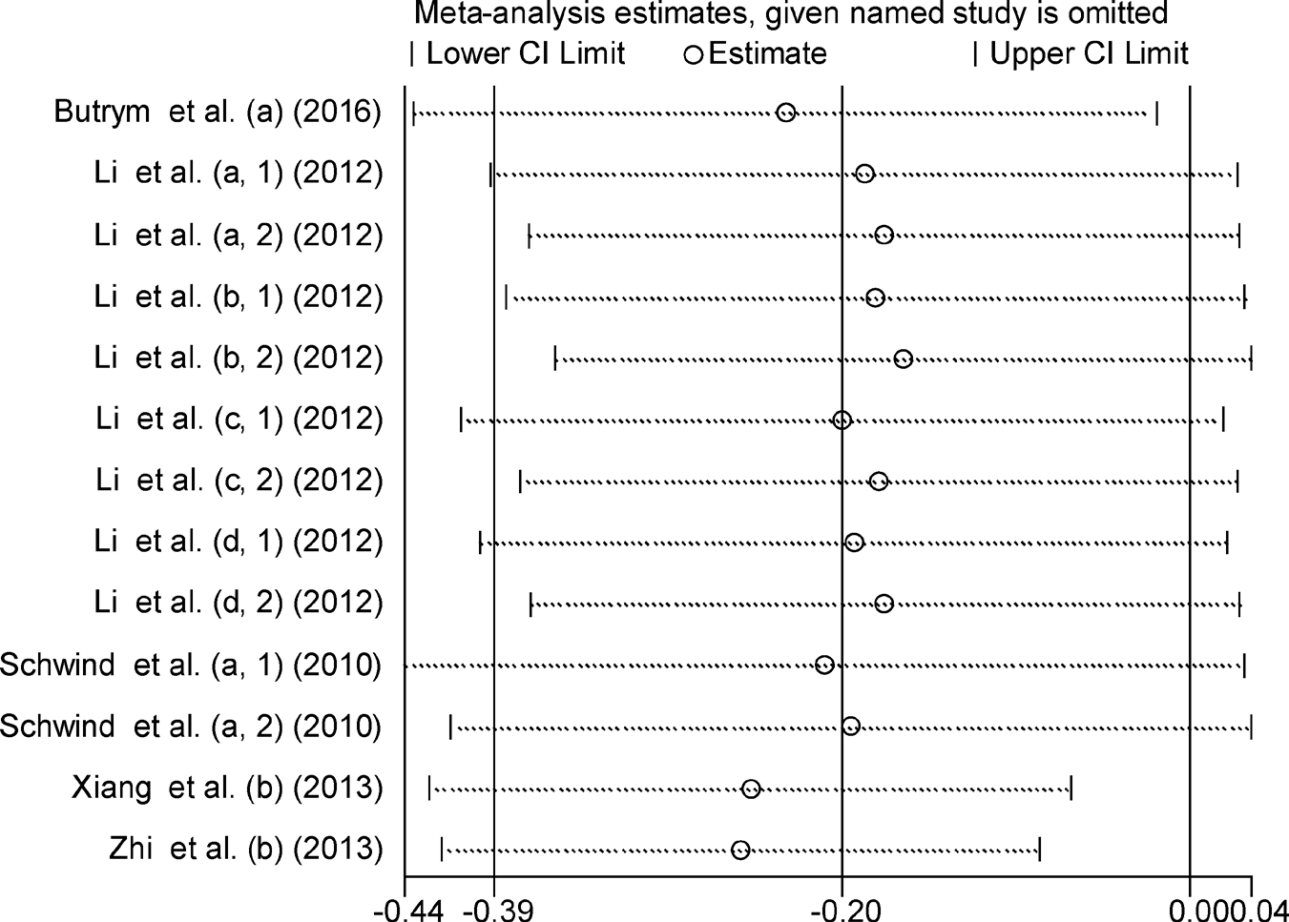
Sensitivity analysis of the relationship between miR-181 expression and OS The lower-case letters a, b, c, d represent that miR-181a, miR-181b, miR-181c and miR-181d were analyzed separately in the studies.

To investigate the effect of individual study on the overall meta-analysis estimate, sensitivity analysis was performed by calculating the pooled HRs with successive exclusion of one study. The result showed that pooled HRs did not change substantially with removal of any study (Figure 4), indicating a more reliable results of this meta-analysis.

### Publication bias

**Figure 5:**
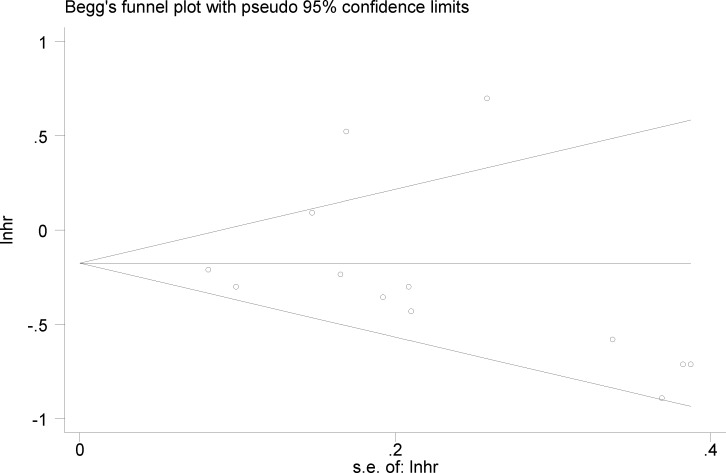
Begg’s funnel plot of miR-181 and OS for publication bias testing

Begg’s and Egger’s tests were used to assess the potential publication bias of the enrolled datasets that evaluated the correlation between OS and miR-181. The *P* values for Begg’s and Egger’s test were 0.127 and 0.680, respectively. Moreover, the funnel plot of the OS analysis based on miR-181 revealed no obvious asymmetry (Figure 5).

## DISCUSSION

Even though accumulating evidence has indicated an association between miR-181 expression and hematological malignancies [37, 38], the prognostic role of miR-181 for survival in AML patients remains controversial. The inconsistent results among published studies could be due to the following factors: diverse origins of population, different platforms of miRNA profiling, and/or relatively small sample size in individual research. Lin et al. reported that miR-181a/b was significantly associated with OS in hematological malignancies (pooled HR: 0.72, 95% CI: 0.63–0.82) [39]. They analyzed hematological malignancies in 2 AML studies in 330 American patients and 2 chronic lymphoid leukemia (CLL) studies in 86 American and 150 Chinese patients. As the heterogeneity was found in AML and CLL, American and Chinese patients, the accuracy of their conclusion requires further confirmation. In our meta-analysis, six published articles were enrolled to evaluate the relationships between all members of miR-181 family and OS, CR, DFS, and RFS in AML patients. Overall, we found a positive correlation between miR-181 expression and survival in American AML patients (*P* < 0.01), with pooled HR of 0.74 (95% CI: 0.67–0.82) for OS, 1.77 (95% CI: 1.25–2.52) for CR, 0.78 (95% CI: 0.67–0.91) for DFS, and a negative correlation between miR-181 expression and survival in Chinese AML patients (*P* < 0.01), with pooled HR of 1.78 (95% CI: 1.35–2.34) for OS, 0.54 (95% CI: 0.34–0.86) for CR, 2.07 (95% CI: 1.41–3.04) for RFS. The result in Polish AML patients still requires further confirmation, as there is only one study evaluating the association between OS and miR-181 (HR: 1.10, 95% CI: 0.82–1.46). The incidence rate of adult is much higher than that of children and adolescent, indicating that different pathogenesis may exist between adult and pediatric AML patients. Since differences are existed between adult and pediatric AML patients, the prognostic values of miR-181 may be inconsistent. Liu et al. detected the expression of miR-181a in pediatric AML patients in China [32], and analyzed the correlation between the level of miR-181a and RFS (HR: 2.15, 95% CI: 1.31–3.54), which was similar with Xiang’s result (HR: 1.96, 95% CI: 1.13–3.72) in Chinese adult AML patients [30]. As there was only one dataset evaluating the prognostic value of miR-181 in pediatric AML patients, the results may require further validation with large datasets. Meanwhile, due to the limited samples size involved in CR, DFS, and RFS, our results may require further validation with large datasets. To our knowledge, this is the first meta-analysis that provides a systemic evaluation of the prognostic value of miR-181 in AML patients.

There are several reported mechanisms supporting the protective role of miR-181 in AML patients. Nanbakhsh et al. indicated that overexpression of miR-181a in AML blasts resulted in the attenuation of their resistance to chemotherapy and NK-cell-mediated killing [40]. In addition, it was found that miR-181a could directly downregulate KRAS, NRAS and MAPK1, resulting in longer survival in a murine AML model treated with miR-181a mimics [41]. Lu et al. demonstrated that miR-181b increased the sensitivity of K562/A02 and HL-60/ADM cells to cytotoxic chemotherapeutic agents and promoted drug-induced apoptosis by inhibiting HMGB1 and MCL-1 expression [42]. Moreover, Bai et al. also proved that overexpression of miR-181a in HL-60/Ara-C cells could induce cytochrome C release and caspase 9/caspase 3 activation by directly targeting BCL-2 [43]. However, Bräuer-Hartmann et al. proved that overexpression of miR-181a/b inhibited granulocytic differentiation by targeting tumor suppressor gene RASSF1A and regulating the cell-cycle regulator cyclin D1 in NB4 cells [37]. Liu et al. found that overexpression of miR-181a increased the ratio of S-phase cells and significantly promoted cell proliferation by directly decreasing the tumor suppressor ataxia telangiectasia mutated (ATM) in NB4 and K562 cells [32]. Su et al. also demonstrated that miR-181 inhibited granulocytic and macrophage-like differentiation of HL-60 cells by directly inhibiting the expression of protein kinase C delta (PRKCD), CTD small phosphatase like (CTDSPL) and calcium/calmodulin dependent protein kinase kinase 1 (CAMKK1) [22]. These results not only highlight the importance of miR-181 as a prognostic biomarker in AML patients, but also indicate that miR-181 may serve as a potential therapeutic target for AML treatment.

Prognostic biomarkers are important for understanding the biological processes of diseases, designing accurate therapeutic strategies, and evaluating the prognosis for patients [44]. There are many prognostic biomarkers in leukemia, including leukocyte morphology and size test in blood, bone marrow or spleen [45], circulating cytokine levels [46], genetic variations [47], and epigenetics characteristics [48]. These distinct (epi)genetic and biological features are also associated with clinical responses to different therapeutic regiments. The clinical outcomes of AML patients are affected by white blood cell count [49], metabolic status [50], and genetic alterations (i.e. chromosomal rearrangements [51] and genetic mutations [52, 53]). For example, Ma et al. found that gene mutations of IDH1, IDH2 and high IDH1 expression were associated with a poor prognosis in CN-AML patients with shorter OS and event-free survival [54]. Chen et al. showed that a panel of 6 serum metabolite markers (including Lactate, 2-Oxoglutarate, Pyruvate) demonstrated prognostic value in CN-AML patients [50]. Recently, researchers have paid increasing attention to epigenetic biomarkers that contribute to the leukemogenesis [55], which include DNA methylation [56] and miRNA dysregulation [26]. The etiology of leukemia is complex, and combinational assessment of different biomarkers is expected to provide more reliable methods to assist medical decision-making and predict the clinical outcomes. In the present study, we confirmed the role of miR-181 in the prognosis of AML patients, but further studies are required to evaluate whether miR-181 influences the patients’ responses to therapeutic regimens in the clinic.

In conclusion, this meta-analysis indicates that higher miR-181 levels are positively associated with the prognostic outcome in American AML patients, and indicate a worse prognostic outcome in Chinese AML patients. Our findings contribute to the better understanding of epigenetic modifiers in tumorigenesis of AML, and pinpoint a novel biomarker and potentially a new therapeutic target in AML patients. We are aware of several limitations in this study. First, a high heterogeneity (*I*^2^ = 73.6%, *P* < 0.001) was found among the datasets evaluating the correlation between OS and miR-181. Sensitivity analysis showed no single study profoundly influenced the stability of the results. However, both subgroup analyses and meta-regression indicated the origin of patients contributed to the heterogeneity. Second, several HRs with 95% CI were extrapolated based on the data extracted from the Kaplan-Meier curves, which could slightly differ from the exact HRs. The samples in these studies were obtained from different tissues (bone marrow, plasma or unknown specimen), and the expression levels of miR-181 were detected by different methods (TaqMan PCR, SYBR Green PCR, or microarray), which might influence the accuracy of miR-181 expression. Moreover, the information of the race and ethnicity of AML patients was unable to be acquired from the enrolled studies, so further studies are required to elucidate the association between miR-181 and patient origin. Finally, the number of studies in sample types and subtype analyses was relatively small, and well-designed investigations with larger sample size are required for future validation of our findings. Furthermore, the role of miR-181 in the outcome of each French, American, and British (FAB) classification and/or cytogenetic subtype is of great interest for future studies.

## MATERIALS AND METHODS

### Search strategy

Published studies were systematically searched in PubMed, Embase and Cochrane Library databases up to May 15, 2017 without language restrictions by two independent researchers (Qiang Guo and Junwen Luan). The following keywords were applied simultaneously: (miR-181* OR miRNA-181* OR microRNA-181* OR miR181* OR miRNA181* OR microRNA181*) AND (“leukemia, myeloid, acute” OR “AML” OR “acute myeloid leukemia” OR “acute myeloid leukaemia”). Moreover, potentially related studies were also collected from the reference lists of the screened full-text articles above.

### Inclusion and exclusion criteria

The eligible studies should follow these inclusive criteria: (1) the study subjects were patients with AML; (2) miR-181 expression levels were measured; (3) the association between a member of miR-181 family and clinical outcome, such as OS, CR, DFS, or RFS, was reported. Studies were excluded if they were: (1) abstracts, meta-analysis, or review articles; (2) without patient samples; (3) nondichotomous miR-181 expression levels; (4) lack of key information such as HR, 95% CI and *P* value. When duplicate publications were identified, only the newest or most informative single article was selected. We also contacted authors of some studies for additional information and data needed for the present meta-analysis. The entire searching was conducted independently by two authors (Qiang Guo and Junwen Luan), and supervised by a third author (Xia Li). Any disagreement was checked and resolved by discussion among the authors.

### Quality assessment

A quality assessment was independently performed for all of the included studies by two authors (Xiaoxiao Zhu and Lin Zhao) using the NOS [33], and any disagreement was resolved by discussion and consensus. The NOS comprises the following three parameters of quality: the selection of the study groups (maximum score is 4), the comparability of the groups (maximum score is 2), and the outcome of interest (maximum score is 3). All of the three parameters were appraised according to the criteria of NOS, with compliance of one NOS standard = 1. The selection score indicates the degree of representativeness of the selected study, the comparability evaluates the comparability of selected studies based on its design or analysis, while the outcome assesses the comprehensiveness of the research contents. The total score is the sum of all three values, and represents the overall quality assessment of the selected paper. The lowest score is 0 and the highest is 9. Studies with a score greater than 5 were considered to be indicative of high-quality studies [34], and were enrolled in the final analysis in the present study.

### Data extraction

The following information was extracted from all eligible studies: (1) publication details: first author’s last name and publication year; (2) characteristics of studied subject: ethnicity/origin, leukemia types, number of patients and specimen used for miR-181 detection; (3) miR-181 assessment methods and the cut-off definition; (4) survival analysis; and (5) HR of elevated miR-181 for OS (CR, DFS, or RFS), as well as their 95% CI and *P* value. If the HRs were not directly reported in the publication, the corresponding authors were contacted for additional data. If only Kaplan-Meier curves were available, Engauge Digitizer version 9.7 was used to extract data from the graphical survival plots, and HRs and 95% CIs were then calculated using the described method [35, 36]. In studies with both univariate analysis and multivariate analysis, HR obtained from multivariate analyses were preferably extracted as inclusion of other variables in the analysis tends to generate more accurate results. The data extraction was performed independently by two authors (Ni Li and Zhen Zhang), with consultation of a third author (Guosheng Jiang) for disagreements.

### Statistical methods

The pooled HR with 95% CI was used to evaluate the correlation of miR-181 expression and the survival outcome of AML patients. The heterogeneity among all included studies was examined using Cochran’s *Q* test and Higgins *I*-square (*I*^2^) statistic. In case of no or moderate heterogeneity (*P* > 0.1 or *I*^2^ < 50%), the fixed-effect model (Mantel-Haenszel test) was applied; otherwise, the random-effects model (Der Simonian and Laird method) was used. Subgroup analysis and meta-regression were carried out to further explore possible explanations for heterogeneity [57]. In addition, Begg’s funnel plot and Egger’s bias were used to evaluate the potential publication bias [58, 59]. If a publication bias did exist, the Duval and Tweedie nonparametric Trim and Fill method was used to adjust the results [60]. Sensitivity analysis was performed by removing one study at each time to assess its influence on the pooled HR. All analyses were conducted by STATA package version 12.0 (Stata Corporation, College Station, Texas, USA). A *P* value < 0.05 was considered to be statistically significant.

## SUPPLEMENTARY MATERIALS TABLE





## Abbreviations

AML: acute myeloid leukemia
ATM: ataxia telangiectasia mutated
CA-AML: cytogenetically abnormal acute myeloid leukemia
CAMKK1: calcium/calmodulin dependent protein kinase kinase 1
CI: confidence interval
CLL: chronic lymphoid leukemia
CN-AML: cytogenetically normal acute myeloid leukemia
CR: complete remission
CTDSPL: CTD small phosphatase like
DFS: disease-free survival
FAB: French, American, and British
FLT3-ITD: FLT3-internal tandem duplication
HR: hazard ratio
miRNA: microRNA
miR-181: microRNA-181
NOS: Newcastle-Ottawa Quality Assessment Scale
OS: overall survival
PRKCD: protein kinase C delta
qPCR: quantitative PCR
RFS: relapse-free survival
SNP: single nucleotide polymorphism
