# Supplementary material for: MicroRNA-181 as a prognostic biomarker for survival in acute myeloid leukemia: a meta-analysis
Source: Oncotarget. 2017 Jul 12;8(51):89130–41. doi: 10.18632/oncotarget.19195 (PMC5687675; doi:10.18632/oncotarget.19195)
Supplement: Supplementary file 1 [file oncotarget-08-89130-s001.pdf]

MicroRNA-181 as a prognostic biomarker for survival in acute myeloid leukemia: a meta-analysis

SUPPLEMENTARY MATERIALS

Supplementary Table 1: Clinical characteristics of AML patients in 6 eligible studies. See Supplementary_Table_1.
