# Supplementary material for: MicroRNA-181 as a prognostic biomarker for survival in acute myeloid leukemia: a meta-analysis
Source: Oncotarget. 2017 Jul 12;8(51):89130–41. doi: 10.18632/oncotarget.19195 (PMC5687675; doi:10.18632/oncotarget.19195)
Supplement: Supplementary file 2 [file oncotarget-08-89130-s002.docx]

Supplementary Table 1: Clinical characteristics of AML patients in 6 eligible studies.

| **Characteristic** | | **Butrym *et al.***  **(2016)** | **Li *et al.***  **(2012)** | | **Liu *et al.***  **(2016)** | **Schwind *et al.***  **(2010)** | **Xiang *et al.***  **(2013)** | **Zhi *et al.***  **(2013)** | |
| --- | --- | --- | --- | --- | --- | --- | --- | --- | --- |
|  |  |  | Training Set | Validation Set |  |  |  | Training Set | Validation Set |
| Gender, n | | 95 | 33 | 53 | 57 | 187 | 158 | 45 | 95 |
|  | Male | 56 | 11 | 27 | 33 | 89 | 71 | 24 | 58 |
|  | Female | 39 | 22 | 26 | 24 | 98 | 87 | 21 | 37 |
| Age (years) | |  |  |  |  |  |  |  |  |
|  | Range | 22-90 | 5-87 | 0-84 |  | 18-59 | 16-60 |  |  |
|  | Median | 61 | 41 | 56 |  | 45 | 42 |  |  |
| Age at diagnosis (years) | |  |  |  |  |  |  |  |  |
|  | <8 |  |  |  | 28 |  |  |  |  |
|  | ≥8 |  |  |  | 29 |  |  |  |  |
| Age (mean±SD) | |  |  |  |  |  |  | 51.42±16.31 | 47.90±15.23 |
|  | <47 |  |  |  |  |  |  | 28 | 48 |
|  | ≥47 |  |  |  |  |  |  | 17 | 47 |
| French-American-British subtype, n | |  |  |  |  |  |  |  |  |
|  | M0 | 7 | 0 | 1 |  |  | 1 |  |  |
|  | M1 |  | 2 | 4 | 4 |  | 22 | 5 | 14 |
|  | M2 |  | 5 | 8 | 23 |  | 66 | 13 | 33 |
|  | M1+M2 | 63 |  |  |  | 92 |  |  |  |
|  | M3 |  | 8 | 9 | 14 |  |  | 8 | 9 |
|  | M4 |  | 8 | 11 | 6 |  | 23 | 8 | 10 |
|  | M5 |  | 8 | 7 | 10 |  | 38 | 11 | 29 |
|  | M4+M5 | 25 |  |  |  | 56 |  |  |  |
|  | M6 |  | 0 | 0 |  |  | 8 |  |  |
|  | Not determined |  | 2 | 13 |  |  |  |  |  |
| Mutation status, n | | 60 |  |  |  |  |  |  |  |
|  | AML-ETO (positive/negative) | 4/56 |  |  | 10/47 |  |  |  |  |
|  | CEBPA (mutated/wild type) |  |  |  |  | 32/152 |  |  |  |
|  | CBFβ-MYH11 (positive/negative) | 2/58 |  |  | 6/41 |  |  |  |  |
|  | FLT3-ITD (positive/negative) | 13/47 |  |  |  | 70/117 | 43/115 |  |  |
|  | FLT3-TKD (positive/negative) |  |  |  |  | 18/167 |  |  |  |
|  | IDH1 (mutated/wild type) |  |  |  |  | 19/124 |  |  |  |
|  | IDH2 (mutated/wild type) |  |  |  |  | 17/126 |  |  |  |
|  | MLL (positive/negative) |  |  |  | 4/53 |  |  |  |  |
|  | MLL-PTD (positive/negative) |  |  |  |  | 12/175 |  |  |  |
|  | NPM1 (positive/negative) | 7/53 |  |  |  | 120/67 | 47/111 |  |  |
|  | PML-RARα (positive/negative) |  |  |  | 9/48 |  |  |  |  |
|  | WT1 (mutated/wild type |  |  |  |  | 22/161 |  |  |  |
| Cytogenetic abnormalities, n | |  |  |  |  |  |  |  |  |
|  | t(8;21) |  | 7 | 9 |  |  |  |  |  |
|  | t(11q23) |  | 15 | 10 |  |  |  |  |  |
|  | t(15;17) |  | 7 | 9 |  |  |  |  |  |
|  | inv(16)/t(16;16) |  | 4 | 9 |  |  |  |  |  |
|  | +8 |  | 0 | 10 |  |  |  |  |  |
|  | -7/del(7q) |  | 0 | 2 |  |  |  |  |  |
|  | Other abnormal karyotype |  | 0 | 4 |  |  |  |  |  |
